# The population genomics of yellowfin tuna (*Thunnus albacares*) at global geographic scale challenges current stock delineation

**DOI:** 10.1038/s41598-018-32331-3

**Published:** 2018-09-17

**Authors:** Carlo Pecoraro, Massimiliano Babbucci, Rafaella Franch, Ciro Rico, Chiara Papetti, Emmanuel Chassot, Nathalie Bodin, Alessia Cariani, Luca Bargelloni, Fausto Tinti

**Affiliations:** 1 Department Biological, Geological and Environmental Sciences (BIGEA), University of Bologna, Via Selmi 3, 40126 Bologna, Italy; 2 Institut de Recherche pour le Développement (IRD), UMR MARBEC (IRD/Ifremer/UM2/CNRS) SFA, Fishing Port, BP570 Victoria, Seychelles; 3 Comparative Biomedicine and Food Science, University of Padova, viale dell’Università 16, 35020 Legnaro, PD Italy; 4 School of Marine Studies, Molecular Analytics Laboratory (MOANA-LAB), Faculty of Science Technology and Environment, The University of the South Pacific, Laucala Campus, Suva, Fiji; 5 Estación Biológica de Doñana, Consejo Superior de Investigaciones Científicas (EBD, CSIC), c/Américo Vespucio s/n, Sevilla, 41092 Spain; 6 Department of Biology, University of Padova, 35121 Padova, Italy; 7 Berlin Center for Genomics in Biodiversity Research (BeGenDiv), Berlin, Germany

## Abstract

Yellowfin tuna, *Thunnus albacares*, is one of the most important seafood commodities in the world. Despite its great biological and economic importance, conflicting evidence arises from classical genetic and tagging studies concerning the yellowfin tuna population structure at local and global oceanic scales. Access to more powerful and cost effective genetic tools would represent the first step towards resolving the population structure of yellowfin tuna across its distribution range. Using a panel of 939 neutral Single Nucleotide Polymorphisms (SNPs), and the most comprehensive data set of yellowfin samples available so far, we found genetic differentiation among the Atlantic, Indian and Pacific oceans. The genetic stock structure analysis carried out with 33 outlier SNPs, putatively under selection, identified discrete populations within the Pacific Ocean and, for the first time, also within the Atlantic Ocean. Stock assessment approaches that consider genetic differences at neutral and adaptive genomic loci should be routinely implemented to check the status of the yellowfin tuna, prevent illegal trade, and develop more sustainable management measures.

## Introduction

Tunas are highly valued species. The tuna fishing industry accounts for up to 8% of all fish and shellfish products in international seafood markets. The large demand from international markets and the overcapacity of the global tuna fleet have led to the overexploitation of many stocks. Thirty-seven percent of tuna stocks worldwide are fully exploited, while 33.5% are already overexploited^1^.

Despite the growing evidence that ignoring population genetic structure in fisheries management may result in local depletion through overexploitation^2^, data on genetic differentiation of tuna species were not yet fully incorporated in the assessment process for most tuna Regional Management Fisheries Organisations (tRMFOs)^3–5^. Information on tuna population structure gathered by recent more comprehensive sampling and more powerful genomic approaches offer the possibility that RFMOs may need to revise previous perceptions of stock structure. The misspecification of genetically different populations may lead to management that negatively affects productivity and long-term stability of tuna populations, as well as the capacity of populations to respond to the variability of oceanic conditions and fishing pressures^5,6^. Designing stock-specific management plans and conservation policies tailored to life history traits, genetic structure and ecological dynamics would mitigate the risk of stock decline and irreversible collapse^1,7,8^.

Yellowfin tuna (*Thunnus albacares*, YFT) is among the most valued wild-caught fishes of the world^9^. Over the last 60 years, YFT have been caught across large areas of the Pacific, Indian and Atlantic Oceans, with the majority of catch coming from the inter-tropical zone^5^. YFT is patchily distributed in all oceans and seas in tropical and temperate latitudes between 45° north and south of the equator^9^. YFT carries out feeding migrations across immense oceanic distances to search for prey to support its high metabolic rates^5^.

The high dispersal potential of YFT led to the initial expectation of a globally panmictic population^10^. This hypothesis was supported by the assumption that oceanic-scale currents facilitate high connectivity among Atlantic, Pacific and Indian demes. This mainly stems from the fact that YFT displays life history traits and ecological characteristics (i.e. high fecundity and large population size) that make it difficult to detect fine-scale genetic differentiation among populations by analysing only a few individuals and a small number of neutral loci (i.e. microsatellites, mitochondrial DNA) representative of a limited portion of the whole genetic variability^5,7,11,12^. The recent introduction of genome-wide Single Nucleotide Polymorphism (SNP) genotyping has increased the accuracy and speed of population genetic analyses at relatively low costs. In the case of YFT, the application of SNP markers has already shown that genetically divergent populations occur at local and oceanic scales^3,13^.

The separation of SNPs that are potentially under-selection (so-called outlier loci) from those that are purportedly neutral in the YFT has allowed for the investigation of genomic regions putatively influenced by selective processes. For the first time, Grewe *et al*.^3^ showed that YFT samples from the Western, central and eastern Pacific are genetically differentiated at putatively adaptive loci. Furthermore, in a recent study, Barth *et al*.^11^ assessed population samples from across the globe and hypothesised that local adaptation or selection are acting on some regions of the YFT genome. Understanding how genome-environment interactions modulate the distribution of neutral and adaptive genetic diversity within and between populations and species is a major challenge of modern biology^14^. The importance of this knowledge comes from the paradigm of local adaptation, and how the fragmentation of the gene pool of a species is translated into a geographical and ecological structure of populations exposed to different selective regimes, resulting in co-adapted gene complexes. Maintenance of the genetic integrity of populations is also essential in a modern perspective of conservation and sustainable use of marine fisheries resources^4^. Understanding adaptive genetic differences between populations can enhance the decision-making process in conservation by identifying priority populations to manage, protect or use for restocking^14^. In addition, this information is the basis for a rational definition of management units that provide a spatially explicit framework necessary for any management plan^14^. The use of Next Generation Sequencing (NGS) technologies to investigate adaptive genomic population structure may reveal a fine scale population structure in tuna species worldwide and shed light on the nature and magnitude of habitat discontinuities or barriers to dispersal that reduce gene flow among populations. It may also inform a more accurate definition of stock boundaries and significantly enhance fisheries management. Finally, the availability of molecular markers that identify the geographical origin of fish landed at international ports may help curb illegal, unregulated and unreported (IUU) fishing^15^.

In the present study, we utilise the enhanced genetic resolution provided by SNP genotyping to better define YFT’s existing biological units at global and oceanic scales and, subsequently, contribute to building a basis for more effective management strategies. This work deals with the generation of a novel 2b-RAD SNP dataset with ten geographical samples of juvenile YFT (mostly of them are in the size range of young-of-the-year, YOY, <60 cm FL, which retain the natal genetic signature), originated from Atlantic, Pacific and Indian oceans (henceforth AO, PO and IO respectively) to fully understand how different micro-evolutionary forces are shaping YFT population structure and dynamics between and within ocean, and to perform a robust population genetic analysis. Our research also aims to disentangle whether selection can affect spatial structuring of YFT populations and this has been addressed by comparatively measuring genetic diversity and population differentiation on neutral and outlier SNP loci. Even if we have the most complete sampling design at the global scale for this species, additional analyses based on a more spatially dense and temporally replicated experimental design are needed to fully uncover the YFT structure within oceans. Our analyses speak in favour of a local/regional population structure within these large stocks linked to adaptive processes.

## Results

### SNP validation and outlier detection

**Figure 1:**
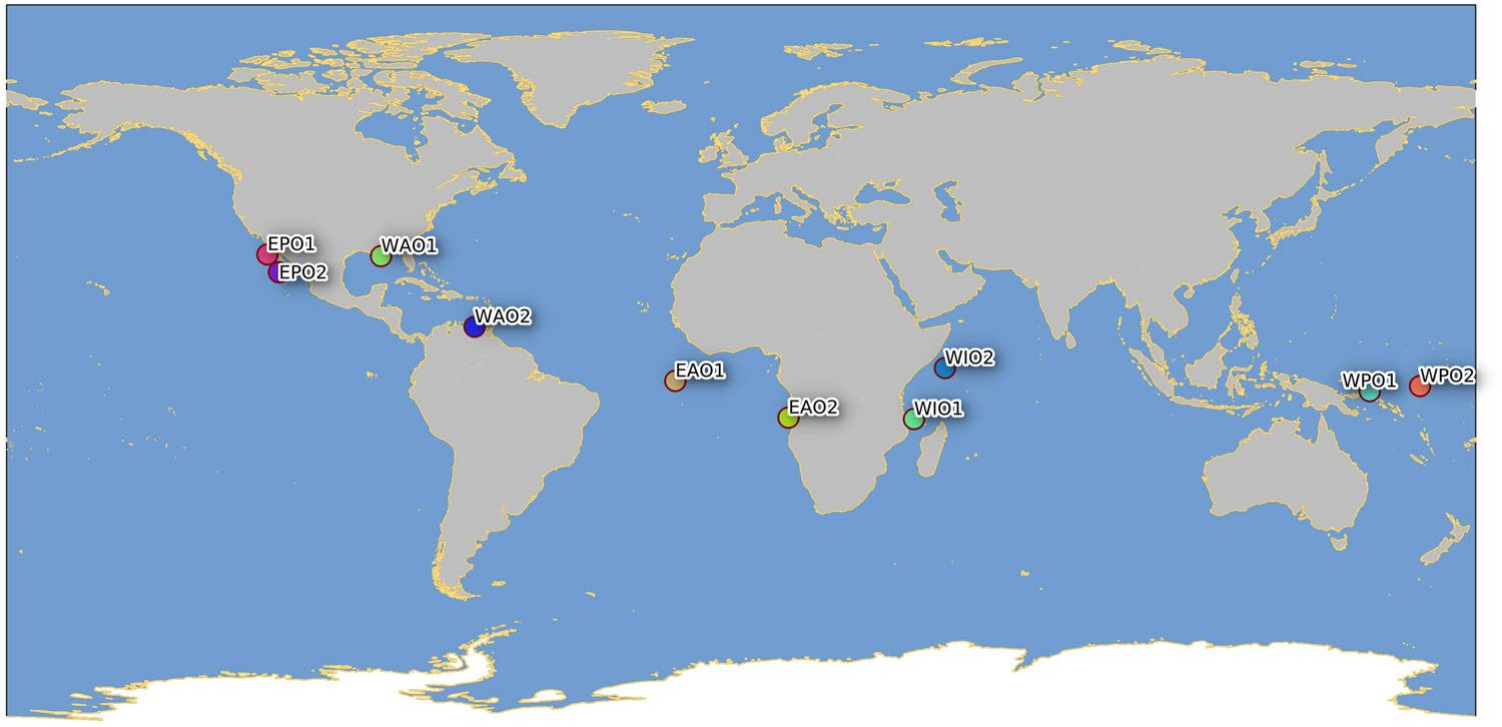
Map of the sampling locations of *Thunnus albacares*. Samples are given as in Table 1. This figure was produced using QGIS.

Libraries using the reduced representation protocol 2b-RAD were successfully obtained for 378 out of 400 juvenile YFT sampled for this study (Fig. 1; Table 1). After quality filtering and removing the adaptors from the sequences, we obtained sequences of 34 base pairs (bp) and on average a total of 2,636,921 reads per specimen from each pool. About 20% of raw reads per pool were lost after the filtering of reads and the removing of adaptors, and eight specimens with less than 500,000 reads were removed from the dataset (Supplementary Table S1).

**Table 1:** Sampling and biological data of *Thunnus albacares* samples.

FAO Fishing Area	Sampling location	Sample ID	Latitude	Longitude	Sample size	Individuals analysed	Tissue	Fishing gear	Fork length (cm)
Area 31 (Atlantic, Western Central)	Venezuela	WAO1	11.14	-65.3	37	29	muscle	Bait boat	29–53
Area 31 (Atlantic, Western Central)	Gulf of Mexico	WAO2	28.44	−88.22	40	36	fin clip	Hook and line	27–47
Area 34 (Atlantic, Eastern Central)	Angola	EAO1	−11.13	11.61	40	37	fin clip	Purse seiner	43–71
Area 34 (Atlantic, Eastern Central)	Sierra Leone	EAO2	−2.14	−16.12	40	39	fin clip	Purse seiner	49–68
Area 51 (Indian Ocean, Western)	Madagascar	WIO1	−11.47	42.38	33	17	fin clip	Purse seiner	44–59
Area 51 (Indian Ocean, Western)	Somalia	WIO2	1.02	49.97	40	40	fin clip	Purse seiner	46–76
Area 71 (Pacific, Western Central)	WC Pacific Ocean	WPO1	−4.64	154.05	45	42	fin clip	Pole and line	34–55
Area 71 (Pacific, Western Central)	WC Pacific Ocean	WPO2	−3.4	166.36	40	38	fin clip	Purse seiner	42–71
Area 77 (Pacific, Eastern Central)	Mexico	EPO1	24.5	−113.25	40	36	muscle	Purse seiner	47–76
Area 77 (Pacific, Eastern Central)	Mexico	EPO2	28.86	−116.06	45	43	muscle	Purse seiner	43–68

Filtered reads for the remaining 370 individuals were successfully mapped against the draft genome of YFT^16^ in 89.34% of cases. The Stacks pipeline returned a total of 6,896 bi-allelic SNPs shared by all 370 specimens. After further filtering (call rate > 80% and MAF > 1%), the dataset was reduced to 986 SNPs shared by 357 specimens. All 986 loci conformed to Hardy-Weinberg equilibrium (HWE) in all samples following Bonferroni correction (α < 0.05). Only 14 loci were found to be in linkage disequilibrium at r^2^ ≥ 0.5, which were removed from the dataset. Ultimately, the final dataset consisted of 357 specimens and 972 SNPs.

The initial filtering process has ensured that most genotyping artefacts were removed to avoid confounding effects on outlier analyses. Detection of outlier SNP loci (i.e. loci putatively under selection) was carried out applying two different software. A total of 85 outliers were detected by using BAYESCAN 2.1, all of which were associated with a positive α–value and q < 0.05, suggesting the presence of directional outliers only. Arlequin recognized 47 candidate outlier loci. For further analyses, outlier loci identified by both methods – i.e. a consensus panel of 33 SNP loci (Supplementary Table S2) – were separated into outlier loci (OL) dataset, while the neutral loci (NL) panel contained the remaining 939 putatively neutral SNPs.

### Genetic diversity and population differentiation

We measured genetic diversity and fixation indices, including heterozygosity, inbreeding and differentiation, for both the NL and OL datasets (Supplementary Table S3). All estimates varied among the samples, but He and Ho values derived from the OL dataset were higher than those obtained with NL. Within each panel of loci, not significant deficits of heterozygous genotypes were shown by all samples. We also determined kinship among all possible pairs of individuals within population samples using COANCESTRY using the NL dataset and found no evidence of relatedness (Mean = −0.28, *P* > 0.05; Supplementary Table S3).

#### Neutral loci analyses

**Figure 2:**
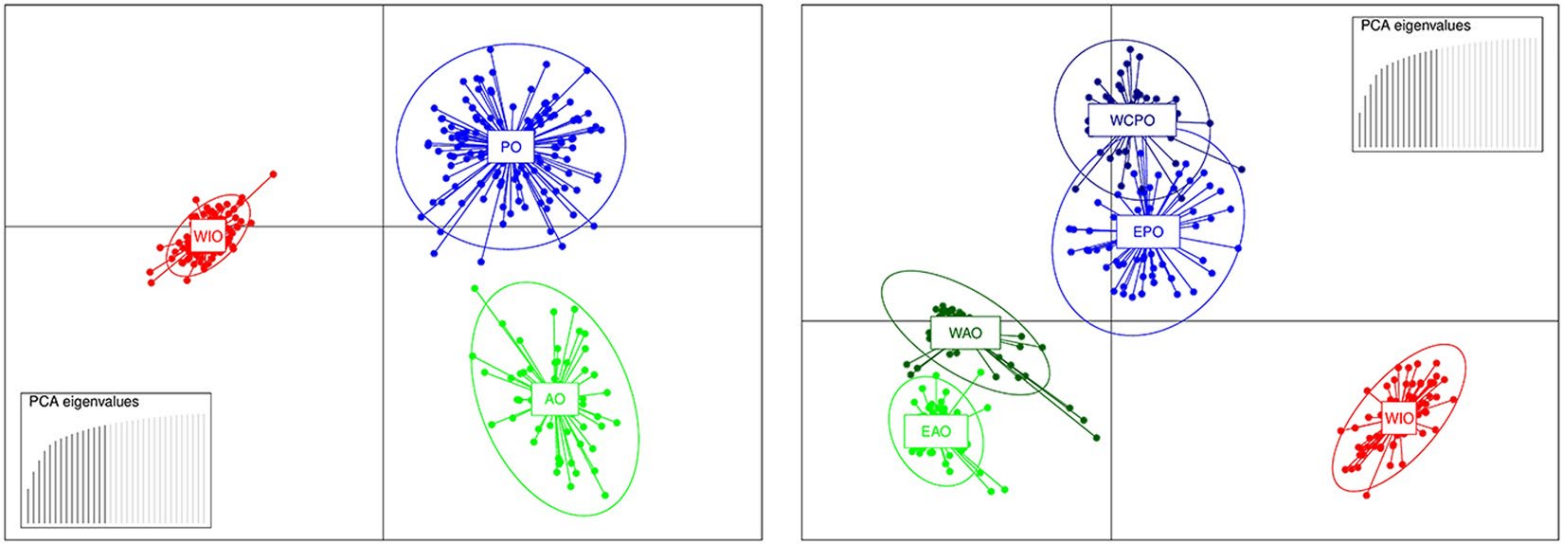
Scatterplots of the DAPC identifying genetic clusters of *Thunnus albacares* using the neutral (NL, **a**) and outlier (OL, **b**) SNP datasets. The individuals are represented as dots and the groups as inertia ellipses. PCA eigenvalues retained in dimension-reduction step of the analysis are displayed in inset, in black. AO: Atlantic Ocean, EAO: Eastern Atlantic Ocean, WAO: Western Atlantic Ocean, PO: Pacific Ocean, EPO: Eastern Pacific Ocean, WCPO: Western-Central Pacific Ocean and WIO: Western Indian Ocean.

**Figure 3:**
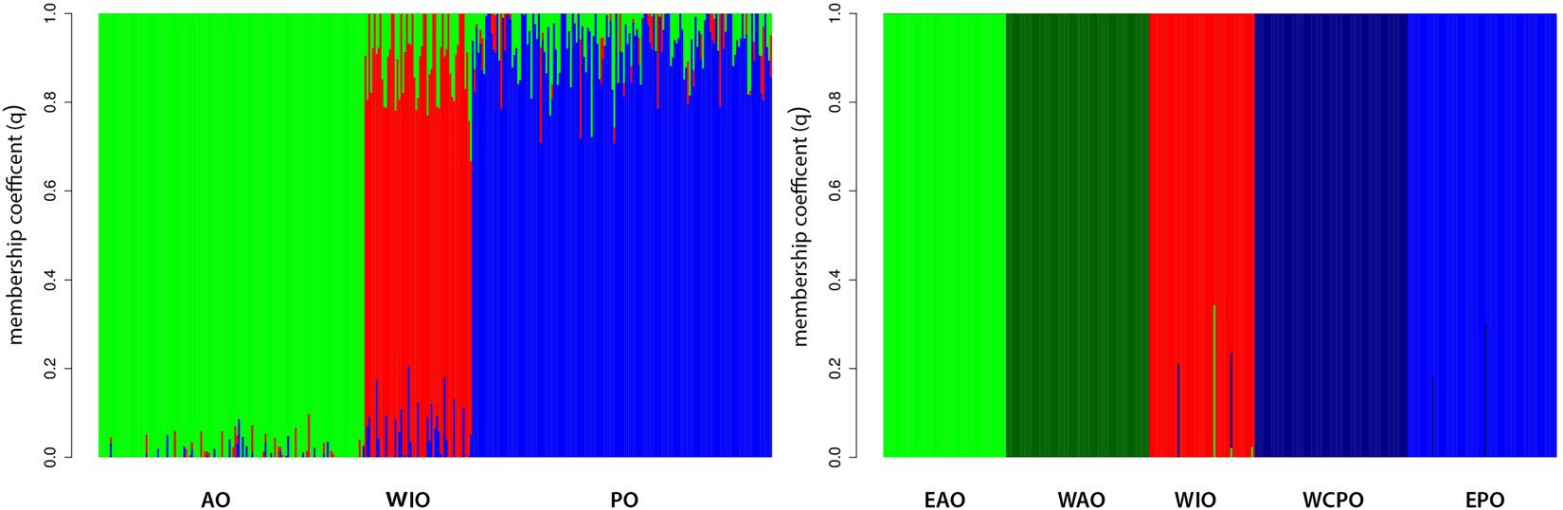
Barplots of the genetic clusters of *Thunnus albacares* identified by FastSTRUCTURE using the neutral (NL, a) and outlier (OL, b) SNP datasets. Each specimen is represented by a vertical coloured line, which is partitioned into K coloured segments. The length of each coloured line is proportional to the estimated membership coefficient (q) from: (**a**) cluster 1 (green, AO), cluster 2 (red, WIO) and cluster 3 (blue, PO); (**b**) 1 (light green, EAO), cluster 2 (dark green, WAO), cluster 3 (red, WIO), cluster 4 (dark blue, WCPO) and cluster 5 (light blue, EPO). Black lines separate different samples based on origin (labelled below the figure). Acronyms are given as in Fig. 2.

A Discriminant Analysis of Principal Components (DAPC) grouped YFT individuals into three well differentiated and not overlapping genetic clusters according to the ocean’s origin (Fig. 2a). The FastSTRUCTURE analysis without *a priori* geographic information confirmed the presence of one cluster per ocean (best K = 3, Fig. 3a) and a clear population subdivision with a small degree of admixture. The AMOVA F-statistics based on clustering of individuals by the three oceans showed that the intra-group genetic variance (F_SC_ = 0.00105) is much lower than the inter-group variance (F_CT_ = 0.08440, Supplementary Table S4). The mean F_ST_ estimate, among these three clusters, based on NL, was 0.08 and significant (P < 0.01). Pairwise *F*_ST_ values among the three clusters were also significantly different from zero (Table 2). All pairwise comparisons between samples from different ocean basins were significantly different, while none of the intra-ocean comparisons were (Supplementary Table S5). We then used GeneClass2 to assign individuals to their genetically most likely population of origin, using a reference dataset created by randomly choosing and pooling 75% of individuals from each ocean. We used a Bayesian approach, applying the Monte Carlo resampling method with 10,000 simulated individuals for multi-locus genotypes, and assuming an equal prior density of allelic frequencies at each locus in each population. To assign each individual to a population, we used a likelihood ratio test comparing the location where the individual was sampled over the highest likelihood value among the reference populations. Using NL, 79 out of 95 simulated genotypes (85.3%) were correctly assigned to the sample of origin. The rate of correct assignment was 87.5% for the AO samples, with two and three individuals incorrectly assigned to the IO and PO groups, respectively. For the WIO, 85.7% of individuals were correctly assigned, with four YFT wrongly assigned to the other two groups, while for the PO, 82.9% of individuals were correctly assigned and the remaining were attributed to the IO.

**Table 2:** Pairwise *F*_*ST*_ estimates based on 939 neutral SNPs (below the diagonal) and associated P-values (above the diagonal) between the three genetic clusters of *Thunnus albacares* detected as in Figs 2a and 3a.

	AO	IO	PO
AO		<0.01	<0.01
IO	0.09		<0.01
PO	0.13	0.04	

All P-values are given after the Bonferroni standard correction (nominal significant threshold α = 0.01).

#### Outlier loci analyses

The DAPC analysis based on the 33 OL grouped the 357 YFT individuals into five clusters: two in the AO, two in the PO, and one in the IO (Fig. 2b). The FastSTRUCTURE analysis further confirmed the DAPC results by recommending a K of 5 clusters to best explain the samples’ subdivision (Fig. 3b). The AMOVA for the OL dataset likewise showed that a five-cluster system minimised the intra-group genetic variance (F_SC_ = −0.00728) and maximised the variance among groups (F_CT_ = 0.35638; Supplementary Table S4). The overall F_ST_ estimate based on OL and on the five clusters detected using FastSTRUCTURE and DAPC analysis, was almost twice the NL estimate after Bonferroni correction (F_ST_ = 0.15, p < 0.01; Table 3). The within-ocean pairwise F_ST_ comparisons between the eastern and western samples of the AO and PO were similar to the inter-oceanic estimates and highly significant after the Bonferroni correction (α = 0.01; Supplementary Table S5). Yet the GeneClass2 analysis using the OL dataset and a five-cluster format showed that the overall percentage of simulated genotypes correctly assigned to the population of origin increased to 95.7%.

**Table 3:** Pairwise *F*_*ST*_ estimates based on 33 outlier SNPs (below the diagonal) and associated P-values (above the diagonal) between the five genetic clusters of *Thunnus albacares* detected as in Figs 2b and 3b.

	WAO	EAO	WIO	WPO	EPO
WAO		<0.01	<0.01	<0.01	<0.01
EAO	0.22		<0.01	<0.01	<0.01
WIO	0.36	0.38		<0.01	<0.01
WPO	0.32	0.39	0.24		<0.01
EPO	0.34	0.42	0.28	0.25	

All P-values are given after the Bonferroni standard correction (nominal significant threshold α = 0.01).

## Discussion

In this study, we provide the most comprehensive and updated analysis of the global population structure of YFT using high-throughput SNP genotyping and population samples mostly composed by YOY (FL < 60 cm), i.e. the early life-stage individuals that exhibited limited migratory behaviour and are assumed to be caught close to their spawning areas. With the largest individual/SNP dataset reported to date at a global oceanic scale, and a level of resolving power that has only recently become possible, we detected population differentiation among samples collected from the main YFT fishing areas within the three oceans. We observed that the magnitude and patterns of differentiation varied depending on the type of markers used (i.e. neutral vs. outlier loci). Results based on neutral loci pointed out significant differences among the three oceans but lack of genetic structure within ocean. In contrast, analyses based on the outlier SNPs provided evidence for strong differentiation between eastern and western samples collected within the Atlantic and Pacific oceans. No evidence of genetic structure was detected in the Indian Ocean, possibly because samples were collected only in the western part. While genetic differentiation has already been documented within the Pacific Ocean^3^, our study reports for the first time that western and eastern populations are genetically differentiated within the Atlantic Ocean, based on outlier, putatively adaptive, loci.

The panel of 939 neutral SNPs clearly resolved the existence of significant differentiation among YFT of Atlantic, Indian and Pacific origin, confirming the genetic differentiation pointed out in a preliminary study^17^ based on a smaller sample size and the same SNP genotyping method. The presence of a discrete YFT population unit in each ocean was further substantiated by different multivariate and individual-based analyses (i.e. DAPC and fastSTRUCTURE). This finding agrees with recent evidence of genetic separation of Atlantic and Indo-Pacific stocks, as well as with the occurrence of a third cluster within the Indian Ocean^11^. Based on mtDNA, RFLP, and allozyme variability, Ward *et al*.^18^ had already hypothesised population genetic heterogeneity between and within oceans and proposed a subdivision of YFT into at least four stocks: one in the Atlantic, one in the Indian, and two in the Pacific (i.e. western-central and eastern stocks).

Differentiation among YFT populations from the three oceans suggests that gene flow among oceans is restricted and that dispersal may be limited. This pattern of genetic differentiation may be linked to the complete closure of the Isthmus of Panama to ca. 2.8 Myr ago^19^. After that event, dispersal between the Atlantic and Indo-Pacific Oceans would only be possible by passing through the Cape of Good Hope. However, the strong Benguela Current, which transports cold upwelled water northward along the south-west coast of Africa, is known to act as a potential barrier to gene flow (as reported for the coastal fish *Atractoscion aequidens*^19^). The extent to which this biogeographic barrier affects dispersal of epipelagic fishes, including the YFT is unknown and may vary considerably depending on the life history traits of different taxa. The differentiation depicted in our study confirmed the results gathered by a recent study^11^ suggesting that the Benguela Current plays an important role as a barrier to migration for YFT.

The evidence of within-ocean population genetic structure was recently shown in three different studies that used potentially adaptive SNPs^3^, mitochondrial DNA sequences^20^ and neutral microsatellites^21^. Using putatively adaptive loci selected from among sequences obtained by reduced-representation libraries and genotyping-by-sequencing through a proprietary Diversity Arrays Technology protocol, DArTseq™, Grewe *et al*.^3^ showed for the first time that Pacific YFT populations were genetically structured. Our results agree with this pattern of differentiation within the Pacific Ocean putatively driven by selective constraints. However, Grewe *et al*.^3^ described a more complex genetic structure in the Pacific Ocean than that which emerged from our study, with significant differences between samples collected off the west coast of Baja California in Mexico, around Tokelau in the Central Pacific, and within the Coral Sea in Australia. The approximate distance between these samples ranged from 4,000 to 10,000 km, while our western Pacific samples were separated by only 2,000 km. Furthermore, the samples collected in the eastern Pacific were separated by less than 200 km. Therefore, the lack of population genetic differentiation observed in this study comparing to the results obtained by Grewe *et al*.^3^ may simply reflect the geographic proximity of our samples and the lack of samples from the Central Pacific in our study. Our inability to identify a population differentiation at the above scales within the Pacific may also depend on the different number of outlier loci used. We selected 33 outlier loci across the entire geographical range of the species by intersecting the results of two independent detection methods (Arlequin and Bayescan) while Grewe *et al*.^3^ obtained 215 potentially-adaptive SNPs strictly using LOSITAN as a single outlier detection software^22^. Approaches implemented in LOSITAN and Arlequin are based on an island model that uses a coalescent F_ST_-outlier method to assess the distribution of F_ST_ as a function of the heterozygosity. However, the outlier detection method implemented in Arlequin adds the option for hierarchical clustering. In contrast, Bayescan^23^ uses a logistic regression model which explains the observed pattern of diversity by dividing it into locus- and population-specific components. Using multiple methods to detect outlier loci and intersecting results limits the number of markers identified but can guarantee a more conservative approach and reduces Type I error due to false positives^24^. At any rate, additional analyses based on a more spatially dense and temporally replicated sampling design in the whole Pacific Ocean combined with a wider panel of outlier SNPs, are needed to accurately uncover the YFT population genetic structure that tRFMOs can confidently reference to support changes in their stock assessment and management policies, and in turn to achieve fishery sustainability. Furthermore, given the highly migratory nature of tunas, samples from locations separated by hundreds or a few thousands of kilometres should be collected within a few days or weeks to each other to prevent sampling duplication.

In this study, we were not able to obtain samples from the eastern Indian Ocean and, therefore, we cannot verify the presence of additional clusters in this area. The IOTC–2015–SC18–ES04 resolution recently recognised the need for additional studies in the Indian Ocean focusing on areas of admixture at a much finer scale than reported here. The resolution recommends employing multidisciplinary approaches, including population genomic structure assessments together with other population identification methodologies. Nevertheless, the lack of genetic heterogeneity found in this study in the western Indian ocean is not in conflict with those obtained by the largest tagging program ever realised in the Indian ocean by IOTTC from 2005 to 2009, which indicated the possible presence of a single, well-mixed YFT population in the entire basin^25^.

Recent studies, starting with Grewe *et al*.^3^, have demonstrated that determining the spatial distribution of adaptive genetic variation is fundamental to uncovering population structure of recently-diverged, large-sized and geographically connected demes which otherwise may appear panmictic at neutral loci. The effects of selection might indeed influence the population structure much more than genetic drift in species such as the YFT, which has a large effective population size and high dispersal potential^26^. The analysis of outlier loci can help to identify genomic regions associated with ecotype divergence over very recent (ecological) timescales^24^. For instance, some studies have reported that local adaptation in fish populations was related to the occurrence of linked alleles (e.g. in the form of chromosomal rearrangements associated with life-history traits^11^). These alleles, in turn, may interact to mould and define the population structure of YFT. The combination of these evolutionary and biological processes may influence YFT population structure among the three oceans, as well as at the within-ocean scale. Biogeographic and physical barriers to dispersal provide simple and effective obstacles to inter-oceanic movement of fish, but less-effective barriers such as oceanographic fronts (e.g.^27^) and environmental gradients may also be relevant^15^. However, the genetic separation detected in the present study may also be linked to a wide variety of evolutionary and biological mechanisms that promote local adaptation to specific environmental constraints.

Migration behaviours play an important role in shaping population structure and adaptive divergence in highly-migratory large epipelagic fish (i.e. the Atlantic bluefin tuna) with genetic differentiation among populations linked to strong fidelity to natal spawning areas and spatial learning for feeding, with population admixture occurring primarily in the large foraging grounds^28–30^. Unfortunately, such a multidisciplinary and integrated investigative approach, with massive sampling carried out over several years (as done for the Atlantic Bluefin tuna by the ICCAT-GBYP) is still lacking for YFT. Tagging studies have revealed a variety of migration patterns with different management implications. For instance, restricted movements and a high degree of fidelity were displayed by YFT within the Central and Western Pacific^31,32^ and within the Eastern Pacific^33,34^, suggesting a limited mixing over large regional areas. Meanwhile in the Atlantic, YFT juveniles are stationed along the African coast within their main spawning ground in the Gulf of Guinea until they reach the pre-adult stage^35^. Once in the pre-adult stage, they migrate to feeding grounds in the western part of the Atlantic Ocean and return to the Gulf of Guinea to spawn when they reach at least 110 cm fork length and about 3 years of age^36^. Tagging studies have also validated that YFT are able to undertake long-distance horizontal migrations in the Atlantic Ocean, with the individuals tagged in the Gulf of Guinea and in the Africa - Canary Islands region (juveniles and pre-adults) migrating relatively close to the coast, both north to south and vice versa^36^. Likewise, pre-adult and adult YFT tagged in the US waters displayed both trans-Atlantic migrations towards the Gulf of Guinea and towards the Caribbean Sea^37^. Such migratory behaviour of YFT is compatible with movements of individuals at different stages between reproductive areas (e.g. Gulf of Guinea and Caribbean/Gulf of Mexico) and foraging grounds (coastal waters of Maine), as already shown for other Atlantic tuna species targeted by wide tagging programs^38,39^. Therefore, it is possible that most of the sexually mature and maturing YFT individuals migrate back from the north-western Atlantic feeding grounds to the Gulf of Mexico and south-eastern Caribbean for reproduction^37^, inducing the central western Atlantic population to be strongly genetically differentiated from that of the eastern part, as indicated in the present study by using the OL dataset. It is likely that selective constraints and local adaptation in the eastern and western reproductive areas overcome the genetically homogenising effect at the neutral loci given by a sufficient number of migrants per generation exchanged between the populations reproducing in the two areas. Such sufficient level of gene flow between the two Central Atlantic populations is supported by evidence of transoceanic migrations of YFT between the Caribbean and Gulf of Guinea^37^. Even though, there is still not enough tagging and reproductive information to confirm the nature and the rate of the YFT spawning migrations or natal homing across the Atlantic Ocean, or to independently ascertain whether populations from the Gulf of Guinea and the Gulf of Mexico/south-eastern Caribbean are two reproductively isolated units^37^, our results suggest a strong adaptive genetic divergence between the eastern and western stocks. Moreover, discrepancies between our results and much of the findings of previous studies may also be due to sampling design and their use of older fish that migrated to mixed feeding areas. Quite the opposite, we collected juveniles YFT that are characterised by a limited swimming ability and are assumed to be caught close to their spawning areas, which better reflect the true genetic composition of the spawning populations.

### Implications for management and conservation

In the present study, we successfully used potentially adaptive and neutral loci to trace back YFT individuals sampled in different geographical areas of Atlantic, Indian and Pacific Oceans. Our results suggest a possible metapopulation structure in YFT, with different populations occupying various habitat patches that are connected to one another by the movement of individuals among them. While we were able to demonstrate more complexity in the global YFT population structure than previously assumed, we also pointed out that it is not sufficient to use a large number of neutral loci and specimens to detect genetic structure in the YFT (e.g.^3^ and this study). Loci putatively under selection are much more able to properly delineate and separate locally adapted subpopulations at regional and local scales, finely tuning the stock structuring in these highly migratory fish species with large populations. Our results call for a re-assessment of the within-ocean YFT stock subdivision by the International Commission for the Conservation of Atlantic Tunas (ICCAT), by using a multi-disciplinary approach that takes advantage of modern genetic technologies and incorporates the use of loci potentially under-selection to detect adaptive divergence in stocks. In fact, genetically differentiated populations that are potentially adapted to regional environmental constraints may experience different productivity regimes and levels of mortality, with direct repercussions on recruitment, growth, and general demographic patterns. The possible presence of genetically structured populations in the western and eastern Atlantic Ocean require scientific and management communities to consider the potential for multiple, independent assessment and management units within the ICCAT jurisdiction. At the least, demographic models developed for assessing the stock status of YFT should include spatial stratification in order to account for the strong signal observed in the genomic studies. More generally, genomic analyses can provide critical information about the delineation of the areas used in spatially-resolved fish population models, which have been shown to have major impacts on scientific advice (e.g.^40^).

Considering the results gathered in the WCPO^3^, our results suggest that different discrete local populations may coexist within the same oceanic basin with complex feeding and reproductive movement dynamics. For this reason, further effort is required to expand the collection of samples from different locations, and from different years, in order to advance the resolution of the YFT population structure in the Atlantic Ocean.

The main risk is the oversimplification of the YFT population boundaries and consequently the failure to achieve current conservation and sustainability goals. Moreover, thanks to their discriminative power, adaptive loci may also be an effective tool to discourage IUU fishing, which is one of the most serious threats to sustainable fisheries^15,41^.

Our results underline once more the need to include fishery-independent data, such as those from population genomics, into the stock assessment models of YFT to ensure its proper and sustainable management. Further massive and deep surveys and studies as the 5-years ICCAT’s Atlantic Ocean Tropical tuna Tagging Programme (https://www.iccat.int/AOTTP/en) are required to understand the reproductive and feeding dynamics of YFT in the Western Atlantic Ocean in order to confirm our findings and to help develop more realistic population dynamics models and effective fisheries management strategies for this species.

## Materials and Methods

### Ethics statement

No specific permits were required for the work described here. Individuals included in the present study were bought from commercial fisheries and they were not subjected to any experimental manipulation. The study was performed in accordance with the EU directive 2010/63/EU and Italian DL 2014/26. The experiments were monitored and carried out by authorized staff to minimise animals’ suffering.

### Sampling and SNP validation

A total of 400 juvenile *T*. *albacares* (size range 27–76 cm) were collected in 2013 and 2014 from ten locations over the entire species distribution (Fig. 1 and Table 1). Most of the YFT individuals are in the size range of YOY (<60 cm FL), which are characterised by a limited swimming ability and are assumed to be caught close to their spawning areas. Sampling was performed directly on-board of fishery vessels, at port of fish landing, or at tuna canneries.

The multiple approaches for collection of specimens have ensured sampling at a very large geographic scale (Fig. 1). From each fish, a portion of skeletal muscle or finclip was excised and immediately stored in 96% molecular grade ethanol and preserved at −20 °C until DNA extraction. Total genomic DNA (gDNA) was extracted from approximately 20 mg of tissue using the commercial Invisorb® Spin Tissue Mini Kit (Invitek, STRATEC Biomedical, Germany) following manufacturer’s instructions. Extracted DNA was suspended in Milli-Q water and concentration and quality determined by both a NanoDrop ND-1000 spectrophotometer (ThermoFisher Scientific, Waltham, Massachusetts, USA) and a Qubit 2.0 Fluorometer (Invitrogen, ThermoFisher Scientific, Waltham, Massachusetts, USA).

A 2b-RAD library was prepared for each sample and for technical replicates following the method described in^17^. Briefly, gDNA was digested with the *CspC*I restriction enzyme and ligated to library-specific adaptors. Individual libraries (identified by specific barcodes) were amplified and pooled into equimolar amounts according to the corresponding barcode. Pooled libraries were sequenced on an Illumina HiSeq2500 platform with a 50 bp single-read module at the Genomix4Life S.r.l. facility (Baronissi, Salerno, Italy), which also performed data demultiplexing. The quality of demultiplexed reads was checked by using FastQC (www.bioinformatics.babraham.ac.uk/projects/fastqc/). The reads were filtered using a custom Perl script as described in^17^. Filtered reads were mapped against the genome of *T*. *albacares*^16^, retaining only uniquely mapped reads and using the program CLC Genomics Workbench v. 5.1 (CLC Bio, applied length fraction = 1.0, similarity fraction = 0.9, all remaining parameters set to default). Mapping results were exported in SAM format and used as input files for refmap_map.pl in the software Stacks v. 1.32 (http://creskolab.uoregon.edu/stacks/^42,43^). The most appropriate Stacks parameters were identified on the mapped technical replicates, as described in^17^. Quality filtering was performed with the process radtags module with default parameters. Only libraries with >500,000 retained reads were kept. 2b-RAD loci containing bi-allelic SNPs in at least 80% of the individuals within each geographic sample were retained and selected to be exported into Genepop format using the Stacks module populations. SNPs with a minimum allele frequency (MAF) smaller than 1% and genotyping rate less than 0.2 were identified with the R package Adegenet 2.0.1 (^44,45^, R version 3.1.2, R Development Core Team, 2014; http://www.r-project.org) and excluded from downstream analyses. Exact test for Hardy-Weinberg equilibrium (HWE) was carried using the function hw.test (R package Pegas^46^). SNPs out of HWE at p < 0.05 in at least nine out of 10 populations were removed. Linkage disequilibrium was tested for each pair of loci with the log likelihood ratio statistic (G-test) using the program Genepop 1.2 (Genepop on the webpage). A Bonferroni correction was applied to the global significance threshold (α = 0.05) when multiple tests were performed.

### Outlier detection

We identified candidate outlier loci (loci that are putatively under selection and involved in adaptive divergence) by the independent approaches implemented in BAYESCAN 2.1^47^ and Arlequin ver. 3.5.1.2^48^. Using BAYESCAN 2.1^47^, we conducted 20 pilot runs of 5,000 iterations each, followed by an additional burn-in of 50,000 iterations and then 5,000 samplings with a thinning interval of 10. To correct for multiple testing, the program computes q-values based on the posterior probability for each locus. Loci with an α-value significantly >0 and q-values < 0.05 were defined as “outliers” – i.e. loci putatively under directional selection. Loci with an α-value significantly <0 were considered putatively under balancing selection. Remaining loci were classified as neutral.

We used a hierarchical Fdist model^49^ implemented in Arlequin ver. 3.5.1.2^48^ as a second approach to detect outlier loci. Loci with significantly higher F_CT_ or F_ST_ values (at the 0.05 significance threshold) were classified as outliers potentially under directional selection among groups. Loci with significantly low F_CT_ or F_ST_ values were classified as putatively under balancing selection. Remaining loci were considered as putatively neutral. Results from the two programs were intersected to build a consensus panel of candidate loci with high probability of being under selection. Only the candidate SNPs jointly identified by both programs were categorized as outliers. All downstream analyses were run independently for the neutral and outlier loci datasets.

### Population diversity statistics and differentiation

We assessed the level of relatedness in fish caught in same area using COANCESTRY^50^, in order to assess if any detected high levels of relatedness should be taken into account when interpreting our population structure results. We used the Wang estimator that performs better than the other estimators when sample sizes are small, and many loci are used^51^. Genetic diversity within samples in terms of observed (Ho) and expected heterozygosity (He) and inbreeding index (F_IS_) was calculated using the software Arlequin ver. 3.5.1.2^48^ and 20,000 permutations. All diversity parameters were calculated within each sample (using a single dataset that included both neutral and outlier loci). All tests aimed at investigating population differentiation were applied to two different datasets, one containing the neutral markers and one with the outlier loci. To estimate the level of differentiation among samples, pairwise and global F_ST_ values were calculated with Arlequin ver. 3.5.1.2^48^, using 20,000 permutations and a significance threshold of α = 0.01.

To determine the number of expected genetic clusters (K) present in the dataset, a discriminant Analysis of Principal Components (DAPC) was performed with Adegenet ver. 2.0.1 (^44,45^ R version 3.1.2, R Development Core Team, 2014; http://www.r-project.org), for both neutral and outlier loci without any *a priori* population definition. The find.clusters () function was initially used to run successive numbers of (K)-means clusters of the individuals, across a range of K = 1–20. We identified the best supported number of clusters through comparison of the Bayesian Information Criterion (BIC) for the different values of K. Prior to running a Discriminant Analysis (DA), an optimum number of principle components (in this case, 40 PCs, which accounted for approximately 81% of the total variation in the data set) was identified using the optim.a.score () function. The DA was then run on the retained principal components using the dapc () function. Finally, after selecting the best number of eigenvalues for the DA analysis, the DAPC results (DAPC scatterplots) were visualized graphically with the scatter.dapc () function.

To reconstruct the genetic ancestry of individuals in relation to their cluster membership, we ran FastSTRUCTURE (^52^; https://rajanil.github.io/fastStructure/) using default parameters, a logistic prior, and K from 1 to 12, with 100 replicate runs for each K. To select the value of K that best explained the subdivision in clusters within our dataset, we ran the python chooseK.py script following^52^. To assess the strength of population subdivision in clusters based on DAPC and FastSTRUCTURE, we performed a hierarchical AMOVA with Arlequin ver. 3.5.1.2^48^ and compared F-statistics for each best K provided by previous clustering analyses.

To estimate the discrimination power of neutral vs. adaptive loci and their ability to trace an individual back to the most likely population of origin, we calculated the assignment probability of each individual’s multi-locus genotype to the three and five population samples groupings, respectively. We employed the software GENECLASS ver. 2.0^53^. Assignment tests require comparison of individuals to baseline genetic information. In this study, all sampled and genotyped individuals were used to generate the baseline data set. We used the leave-one-out procedure and removed the individual to be assigned from the reference data set before performing each assignment analysis. The Monte Carlo resampling algorithm^54^ implemented in Geneclass2 was used to estimate a statistical threshold as suggested by^53^. This algorithm is the most appropriate for this analysis because it considers the sample size of the reference populations. Individuals were considered immigrants when the probability of being assigned to the reference populations was lower than 0.05. Accordingly, with STRUCTURE results, we first performed the analysis with NL dataset and the 3 clusters as reference, then with OL and the 5 clusters at intra-oceans level.

### Accession codes

Sequencing data was deposited in the NCBI-short read archive (SRA) database under the accession number SRP067271.

## Electronic supplementary material

Supplementary Tables S1-S5
