# Supplementary material for: The population genomics of yellowfin tuna (Thunnus albacares) at global geographic scale challenges current stock delineation
Source: Sci Rep. 2018 Sep 17;8:13890. doi: 10.1038/s41598-018-32331-3 (PMC6141456; doi:10.1038/s41598-018-32331-3)
Supplement: Supplementary file 1 — Supplementary Tables S1-S5 [file 41598_2018_32331_MOESM1_ESM.docx]

**Supplementary Information for “The population genomics of yellowfin tuna (*Thunnus albacares*) at global geographic scale challenges current stock delineation”**

Carlo Pecoraro^a,b,g*^, Massimiliano Babbucci^c^, Rafaella Franch ^c^, Ciro Rico^d,e^ , Chiara Papetti^f^, Emmanuel Chassot^b^, Nathalie Bodin^b^, Alessia Cariani^a^, Luca Bargelloni^c^, Fausto Tinti^a^

^a^ Dept. Biological, Geological and Environmental Sciences (BIGEA), University of Bologna, Via Selmi 3, 40126 Bologna, Italy

^b^ Institut de Recherche pour le Développement (IRD), UMR MARBEC (IRD/Ifremer/UM2/CNRS) SFA, Fishing Port, BP570 Victoria, Seychelles

^c^ Comparative Biomedicine and Food Science, University of Padova, viale dell'Università 16, 35020 Legnaro, PD, Italy

^d^ School of Marine Studies, Molecular Analytics Laboratory (MOANA-LAB), Faculty of Science Technology and Environment, The University of the South Pacific, Laucala Campus, Suva, Fiji

^e^ Estación Biológica de Doñana, Consejo Superior de Investigaciones Científicas (EBD, CSIC), c/Américo Vespucio s/n, Sevilla 41092, Spain

^f^ Department of Biology, University of Padova - 35121 Padova, Italy

^g^ Berlin Center for Genomics in Biodiversity Research (BeGenDiv), Berlin, Germany

Correspondence and requests for materials should be addressed to Ca. P. (email: info@physalia-courses.org)

**Supplementary Table S1.** Number of raw reads, number and percentage of retained reads after filtering of the ten population samples of *Thunnus albacares* analysed by 2b-RAD. Sample ID are as in Table 1.

| **Sample ID** | **Raw reads** | **Filtered reads** | **% of reads lost** |
| --- | --- | --- | --- |
| **WA01** | 3,312,337 | 2,526,644 | 24.9 |
| **WA02** | 3,244,529 | 2,713,851 | 17.8 |
| **EA01** | 3,433,692 | 2,670,318 | 34.1 |
| **EA02** | 3,159,125 | 2,481,523 | 23.5 |
| **WI01** | 3,674,435 | 2,683,577 | 27.8 |
| **WI02** | 4,081,030 | 3,527,438 | 14.5 |
| **WP01** | 3,623,456 | 3,160,638 | 13.2 |
| **WP02** | 2,878,098 | 2,363,162 | 19.1 |
| **EP01** | 3,328,447 | 2,950,684 | 17.3 |
| **EP02** | 2,768,277 | 2,464,614 | 18.2 |

**Supplementary Table S2.** List of the 33 outlier loci identified by both Arlequin ver. 3.5.1.2 (Excoffier & Lischer 2010) and BAYESCAN 2.1 (Foll 2012).

**In Arlequin** (left side of the table), per each locus is reported I) the observed heterozygosity between population, ii) the observed FST value, iii) the FST p-value, and iv) 1 - the quantile of the observed FST in the distribution.

**In Bayescan** (right side of the table), per each locus are reported the posterior probability for the model including selection (prob), the logarithm of Posterior Odds to base 10 for the model including selection (log10(PO)) and the corresponding qval, the estimated alpha coefficient indicating the strength and direction of selection (alpha), the FST calculated per each population as the posterior mean using model averaging.

| **Arlequin** | | | | | **Bayescan** | | | | | |
| --- | --- | --- | --- | --- | --- | --- | --- | --- | --- | --- |
| **Locus** | **Obs. Het. BP** | **Obs FST** | **FST P-value** | **1-FST quantile** | **Locus** | **prob** | **log10(PO)** | **qval** | **alpha** | **FST** |
| **734** | 0.61 | 0.54 | 0.00 | 0.00 | **734** | 1.0000 | 1000.0 | 0.00 | 3.11 | 0.35 |
| **486** | 0.56 | 0.50 | 0.00 | 0.00 | **486** | 1.0000 | 1000.0 | 0.00 | 3.23 | 0.37 |
| **800** | 0.55 | 0.49 | 0.00 | 0.00 | **800** | 1.0000 | 1000.0 | 0.00 | 3.45 | 0.40 |
| **584** | 0.12 | 0.41 | 0.00 | 0.00 | **584** | 1.0000 | 1000.0 | 0.00 | 2.32 | 0.23 |
| **826** | 0.50 | 0.37 | 0.00 | 0.00 | **826** | 1.0000 | 1000.0 | 0.00 | 1.86 | 0.17 |
| **373** | 0.57 | 0.35 | 0.00 | 0.00 | **373** | 1.0000 | 1000.0 | 0.00 | 2.14 | 0.21 |
| **352** | 0.56 | 0.32 | 0.01 | 0.01 | **352** | 1.0000 | 1000.0 | 0.00 | 2.02 | 0.19 |
| **582** | 0.09 | 0.31 | 0.00 | 0.00 | **582** | 1.0000 | 1000.0 | 0.00 | 2.03 | 0.20 |
| **68** | 0.56 | 0.28 | 0.02 | 0.02 | **68** | 1.0000 | 1000.0 | 0.00 | 1.89 | 0.18 |
| **263** | 0.49 | 0.28 | 0.00 | 0.00 | **263** | 1.0000 | 1000 | 0.00 | 3.29 | 0.17 |
| **755** | 0.43 | 0.28 | 0.01 | 0.01 | **755** | 1.0000 | 1000.0 | 0.00 | 2.04 | 0.20 |
| **306** | 0.52 | 0.26 | 0.02 | 0.02 | **306** | 1.0000 | 1000.0 | 0.00 | 2.00 | 0.19 |
| **972** | 0.41 | 0.26 | 0.01 | 0.01 | **972** | 1.0000 | 1000.0 | 0.00 | 1.78 | 0.16 |
| **55** | 0.52 | 0.26 | 0.02 | 0.02 | **55** | 1.0000 | 1000.0 | 0.00 | 1.79 | 0.16 |
| **428** | 0.54 | 0.25 | 0.03 | 0.03 | **428** | 1.0000 | 1000.0 | 0.00 | 1.89 | 0.18 |
| **246** | 0.32 | 0.24 | 0.02 | 0.02 | **246** | 1.0000 | 1000.0 | 0.00 | 2.11 | 0.20 |
| **268** | 0.29 | 0.23 | 0.03 | 0.03 | **268** | 1.0000 | 1000.0 | 0.00 | 1.96 | 0.18 |
| **854** | 0.40 | 0.23 | 0.03 | 0.03 | **854** | 1.0000 | 1000.0 | 0.00 | 1.67 | 0.15 |
| **329** | 0.49 | 0.22 | 0.03 | 0.03 | **329** | 1.0000 | 1000.0 | 0.00 | 1.85 | 0.17 |
| **440** | 0.48 | 0.22 | 0.03 | 0.03 | **440** | 1.0000 | 1000.0 | 0.00 | 1.96 | 0.19 |
| **240** | 0.44 | 0.22 | 0.03 | 0.03 | **240** | 1.0000 | 1000.0 | 0.00 | 1.90 | 0.18 |
| **146** | 0.37 | 0.21 | 0.04 | 0.05 | **146** | 1.0000 | 1000.0 | 0.00 | 1.89 | 0.18 |
| **219** | 0.21 | 0.17 | 0.04 | 0.05 | **219** | 1.0000 | 1000.0 | 0.00 | 1.83 | 0.17 |
| **601** | 0.15 | 0.17 | 0.02 | 0.02 | **601** | 1.0000 | 1000.0 | 0.00 | 1.97 | 0.19 |
| **141** | 0.09 | 0.15 | 0.00 | 0.00 | **141** | 1.0000 | 1000.0 | 0.00 | 2.00 | 0.19 |
| **202** | 0.08 | 0.14 | 0.00 | 0.00 | **202** | 0.9916 | 2.072 | 0.00 | 2.05 | 0.10 |
| **837** | 0.07 | 0.12 | 0.00 | 0.00 | **837** | 1.0000 | 1000.0 | 0.00 | 1.86 | 0.17 |
| **650** | 0.47 | 0.09 | 0.00 | 0.00 | **650** | 0.98876 | 1.9445 | 0.00 | 1.18 | 0.10 |
| **635** | 0.11 | 0.08 | 0.00 | 0.00 | **635** | 1.0000 | 1000.0 | 0.00 | 1.50 | 0.16 |
| **313** | 0.07 | 0.08 | 0.00 | 0.00 | **313** | 1.0000 | 1000.0 | 0.00 | 1.97 | 0.19 |
| **251** | 0.04 | 0.06 | 0.00 | 0.00 | **251** | 1.0000 | 1000.0 | 0.00 | 1.87 | 0.08 |
| **864** | 0.04 | 0.05 | 0.00 | 0.00 | **864** | 1.0000 | 1000.0 | 0.00 | 2.78 | 0.09 |
| **346** | 0.12 | 0.04 | 0.00 | 0.00 | **346** | 1.0000 | 1000.0 | 0.00 | 1.75 | 0.11 |

**Supplementary Table S3.** Genetic diversity of the ten population samples of *Thunnus albacares* analysed by 2b-RAD. Per each sample the following information are reported: the number of individual analysed successfully, the relatedness expressed in terms of mean and variance using the Wang estimator, the observed and expected heterozygosity (Ho and He) and fixation index (F_IS_) in the neutral and outlier loci datasets. Sample ID are as in Table 1. NS (not significant).

| **Sample ID** | **Individuals analysed** | **Relatedness**  **Wang** | **Neutral Loci** | | |  | **Outlier loci** | | |
| --- | --- | --- | --- | --- | --- | --- | --- | --- | --- |
|  |  | **Mean & Variance** | **Ho** | **He** | **F_IS_** |  | **Ho** | **He** | **F_IS_** |
| WA01 | 29 | -0.34 (0.01) | 0.17 | 0.21 | 0.08(NS) |  | 0.22 | 0.25 | 0.11(NS) |
| WA02 | 36 | -0.32 (0.02) | 0.17 | 0.23 | 0.12(NS) |  | 0.23 | 0.26 | 0.14(NS) |
| EA01 | 37 | -0.32 (0.03) | 0.24 | 0.28 | 0.06(NS) |  | 0.28 | 0.32 | 0.07(NS) |
| EA02 | 39 | -0.29 (0.03) | 0.18 | 0.22 | 0.07(NS) |  | 0.22 | 0.27 | 0.09(NS) |
| WI01 | 17 | -0.31 (0.01) | 0.16 | 0.21 | 0.11(NS) |  | 0.21 | 0.26 | 0.14(NS) |
| WI02 | 40 | -0.26 (0.03) | 0.18 | 0.23 | 0.08(NS) |  | 0.24 | 0.30 | 0.10(NS) |
| WP01 | 42 | -0.26 (0.01) | 0.19 | 0.23 | 0.07(NS) |  | 0.23 | 0.31 | 0.09(NS) |
| WP02 | 38 | -0.22 (0.01) | 0.22 | 0.24 | 0.06(NS) |  | 0.27 | 0.32 | 0.07(NS) |
| EP01 | 36 | -0.28 (0.00) | 0.21 | 0.25 | 0.11(NS) |  | 0.28 | 0.31 | 0.13(NS) |
| EP02 | 43 | -0.23 (0.01) | 0.15 | 0.18 | 0.07(NS) |  | 0.21 | 0.25 | 0.08(NS) |

**Supplementary Table S4.** Results of the AMOVA based on cluster subdivision of *Thunnus albacares*. F-statistics were obtained with Arlequin ver. 3.5.1.2 (Excoffier and Lischer, 2010). 95% Confidence Intervals are reported in square brackets. P-values are not provided since the optimal group subdivision is based on the clustering output of DAPC and fastSTRUCTURE.

|  | **Neutral loci (3 groups)** | **Outlier loci (5 groups)** |
| --- | --- | --- |
| **F_ST_** | 0.08536 [0.07,0.09] | 0.35170 [0.28; 0.47] |
| **F_SC_** | 0.00105 [-0.00,0.01] | -0.00728 [0.11; 0.21] |
| **F_CT_** | 0.08440 [0.06,0.09] | 0.35638 [0.14; 0.37] |

**Supplementary Table S5.** Pairwise *F_ST_* estimates between the ten population samples of *Thunnus albacares* based on the neutral (below the diagonal) and the outlier loci (above the diagonal). Significant values are in bold and those that remained significant after Bonferroni standard correction are underlined (nominal significant threshold α = 0.01).

|  | WA01 | WA02 | EA01 | EA02 | WI01 | WI02 | WP01 | WP02 | EP01 | EP02 |
| --- | --- | --- | --- | --- | --- | --- | --- | --- | --- | --- |
| WA01 | * | 0.012 | **0.239** | **0.234** | **0.218** | **0.216** | **0.319** | **0.309** | **0.336** | **0.354** |
| WA02 | 0.009 | * | **0.235** | **0.232** | **0.295** | **0.326** | **0.386** | **0.382** | **0.394** | **0.410** |
| EA01 | 0.010 | 0.009 | * | 0.008 | **0.240** | **0.241** | **0.358** | **0.349** | **0.364** | **0.381** |
| EA02 | 0.012 | 0.007 | 0.012 | * | **0.306** | **0.337** | **0.398** | **0.394** | **0.407** | **0.422** |
| WI01 | **0.136** | **0.116** | **0.116** | **0.124** | * | 0.012 | **0.390** | **0.396** | **0.386** | **0.398** |
| WI02 | **0.130** | **0.112** | **0.111** | **0.119** | 0.001 | * | **0.383** | **0.388** | **0.389** | **0.400** |
| WP01 | **0.088** | **0.074** | **0.074** | **0.080** | **0.036** | **0.039** | * | 0.005 | **0.235** | **0.253** |
| WP02 | **0.077** | **0.066** | **0.066** | **0.068** | **0.041** | **0.044** | 0.002 | * | **0.250** | **0.271** |
| EP01 | **0.102** | **0.085** | **0.084** | **0.095** | **0.040** | **0.042** | 0.005 | 0.007 | * | 0.007 |
| EP02 | **0.105** | **0.088** | **0.086** | **0.097** | **0.042** | **0.045** | 0.003 | 0.011 | 0.008 | * |
